# A226 SEX-BASED DISPARITY COMPLICATIONS FOLLOWING LIVER TRANSPLANTATION

**DOI:** 10.1093/jcag/gwab049.225

**Published:** 2022-02-21

**Authors:** N Singh, J Chow, M Ebadi, M Ma, A J Montano-Loza, R Bhanji

**Affiliations:** 1 University of Alberta, Edmonton, AB, Canada; 3 Division of Gastroenterology & Liver Unit, University of Alberta, Edmonton, AB, Canada

## Abstract

**Background:**

Sex-based disparity exists in liver transplantation (LT) with women being disadvantaged at every stage of the process starting from assessment to post transplantation (Bryce et al., 2009). The reasons for this are multifactorial and include biological disparities, psychosocial, and allocation inequalities (Burra et al., 2013).

**Aims:**

The purpose of this study was to identify differences in immediate or long-term complications post-LT by sex.

**Methods:**

We analyzed 702 patients who underwent LT at the University of Alberta from 2002 to 2015. Patients aged < 18 years or requiring a repeat or multivisceral transplant were excluded. Renal dysfunction was defined according to the KDIGO criteria. Cardiovascular disease (CVD) was defined as hospitalization for or death from coronary artery disease, cardiac arrest or cerebrovascular disease.

**Results:**

Male patients comprised 69% of the population. Time on the waitlist was similar for men (9.3 ± 11.7 months) and women (9.9 ± 12.3 months; *p*=0.57). Both sexes were comparable in age (males: 53 ± 10 years; females: 52 ± 11 years; p=0.19), MELD (males: 18 ± 9; females: 19 ± 10; p=0.16) and BMI (males: 27.7 ± 5.7 kg/m^2^; females: 27.3 ± 6.6 kg/m^2^; p=0.58). Women had lower creatinine pre-LT (males: 1.1 ± 0.60 mg/dL; females 0.96 ± 0.51 mg/dL; *p*<0.01). There were no differences in donor age, sex or BMI. Women had significantly longer hospital length of stay (males: 18 days [IQR: 11, 32]; females: 25 days [IQR: 14, 43]; p <0.001). There was no difference in risk of acute kidney injury (OR 1.4 [95% CI: 0.98, 2.1]; p=0.06), infection (OR 1.1 [95% CI: 0.8, 1.5]; p=0.52) or rejection episodes (OR 1.1 [95% CI: 0.8, 1.5]; p=0.74) following LT. Women had a higher risk of CKD post-LT (OR 2.3 [95% CI: 1.6, 3.2]; p<0.0001). There was no difference in de-novo diabetes (males: 22%; females: 16%; *p*=0.10), hypertension (males: 45%; females: 48%; *p*=0.41), dyslipidemia (males: 37%; females: 39%; *p*=0.67) and CVD (males: 20%; females: 19%; *p*=0.84) post-LT. Graft (males: 11.4 ± 0.4 years; females: 11.8 ± 0.5 years; p=0.32) and patient survival (males: 11.8 ± 0.4 years; females: 12.4 ± 0.5 years; p=0.18) were similar over a median follow up of 6.3 years [IQR: 3.5, 9.9].

**Conclusions:**

Women spend a longer time in hospital and are at an increased risk of CKD following LT. Despite these differences, overall graft and patient survival are comparable. Our data suggest the disparity between sexes likely exists pre-LT and females that undergo LT have similar outcomes to their male counterparts.

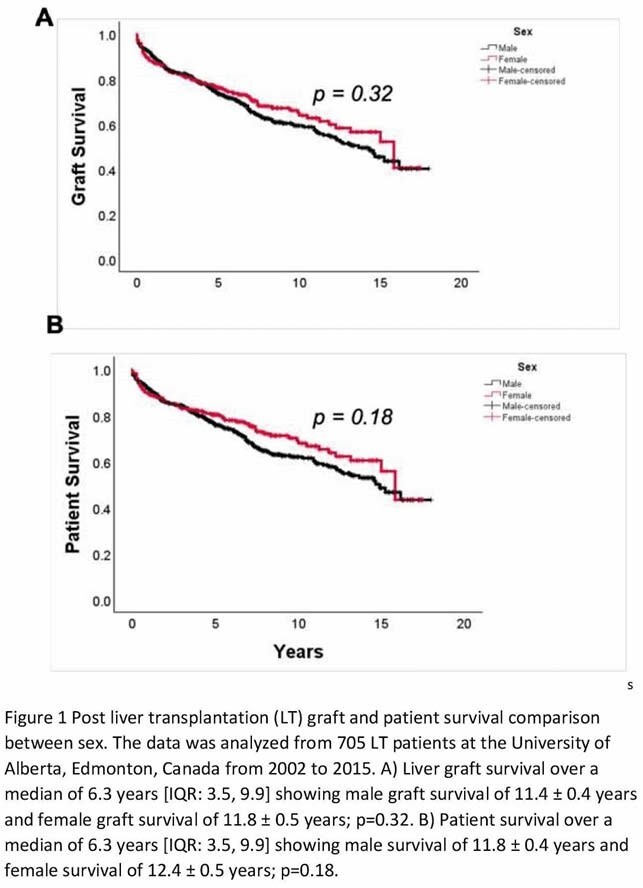

**Funding Agencies:**

None

